# Tire soil reinforcement for slope stability improvement in the PK3 + 400 landslide Medea Algeria

**DOI:** 10.1038/s41598-025-24402-z

**Published:** 2025-11-19

**Authors:** Zohra Melaz, Lyazid Guechi, Saad Saghir, Aissa Cheriet

**Affiliations:** 1 Department of Civil Engineering, Faculty of Technology, University Ferhat ABBAS-Setif1, Setif, Algeria; 2 Civil Engineering Research Laboratory of Setif (LRGCS), Setif, Algeria; 3 Civil Engineering Materials and Environment Laboratory, Ecole Nationale Polytechnique d El-Harrach-Algiers, Algiers, Algeria

**Keywords:** Landslide, Slope stability, Geo-Slope, Tire–soil reinforcement, Sustainable geotechnics, Engineering, Environmental sciences, Natural hazards

## Abstract

Landslides are among the most damaging natural hazards, threatening infrastructure and communities. This study investigates a landslide in Medea, Algeria, located between National Roads 01 and 18, where slope instability disrupts local transport. Geological and geotechnical surveys revealed decompressed marly clays, groundwater infiltration, and seismic activity as the main triggering factors. Stability analyses using Geo-Slope software showed that the slope is stable in dry conditions but becomes unstable when saturated or subjected to seismic loads. To improve safety, a reinforcement technic using recycled tires mixed with soil was evaluated. Numerical simulations confirm that tire–soil reinforcement increases shear strength and raises safety factors above critical thresholds. This sustainable and cost-effective solution demonstrates the potential of recycled materials for slope stabilization and landslide risk reduction.

## Introduction

Slope stability analysis is a fundamental aspect of geotechnical engineering, essential for evaluating and ensuring the safety of slopes under diverse environmental and loading conditions^1^. Slope stability analysis is a fundamental aspect of geotechnical engineering, essential for evaluating and ensuring the safety of slopes under diverse environmental and loading conditions^2^. The assessment of slope stability faces challenges from several uncertainty sources: variability in geotechnical parameters, model limitations, and unpredictable triggers like rainfall and seismic events. The uncertainty level directly relates to site investigation quality and laboratory testing thoroughness. An effective solution requires a robust framework that integrates advanced modeling with comprehensive data collection. This approach improves stability prediction accuracy and enables the development of superior risk mitigation strategies^3^. To address them effectively, it is necessary to adopt a robust framework that combines advanced modeling techniques with comprehensive data collection methods^4^. Such an approach enhances the predictive accuracy of stability assessments and supports the development of more effective risk mitigation strategies^5^.

A major contributor to uncertainty in slope stability evaluation is the spatial variability of soil and rock properties^19^. Even with detailed geotechnical investigations, achieving a complete representation of subsurface conditions is rarely possible without incurring significant costs. Landslide risk has therefore received increasing attention from the scientific community. Slope stability problems are inherently statically indeterminate, requiring assumptions about internal force distributions to estimate the factor of safety (FoS)^20^. Widely used analytical methods include Janbu’s simplified method^6^, Bishop’s simplified method, and the Morgenstern–Price method^7^. These methods are classified as either “simplified,” satisfying only force or moment equilibrium, or “rigorous,” accounting for both. FoS values derived from moment equilibrium are generally considered more reliable, although true equilibrium also requires satisfying force equilibrium^8^.

In parallel, tire–soil interaction has emerged as a relevant research area, particularly in off-road vehicle engineering, due to its influence on traction, mobility, and ride performance. Studies indicate that radial ply tires generally exhibit lower rolling resistance than diagonal ply designs^9^. The tire–soil contact area controls stress distribution within the soil, directly affecting overall performance^10,11^.

Tire–soil reinforcement presents several innovative and sustainable advantages over conventional slope stabilization methods, such as concrete retaining walls or metal anchors. It utilizes recycled tires, thereby reducing environmental impact and promoting waste valorization. Installation is rapid and cost-effective, requiring less labor and fewer expensive materials. Numerical simulations show that this technique significantly increases soil shear strength and slope safety factors, often comparable to traditional solutions. The tire-soil reinforcement adapts well to various soil types. It withstands repeated pressure effectively. This makes it ideal for embankments affected by weather changes or human activities.

In sustainable geotechnical engineering, tire-based reinforcement has gained attention as a cost-effective and environmentally friendly method to improve slope stability. Previous studies have shown that incorporating tire aggregates or fibers into soil can increase shear strength and enhance the FoS^12,13^. For instance, researcher reported that adding 5–10% tire aggregates to clay soils improved shear strength by up to 20%, while other researchers observed that slopes reinforced with tire fibers exhibited reduced depth and velocity of landslides.

Building on this context, the present study evaluates the efficiency of tire–soil reinforcement for stabilizing the PK3 + 400 slope in Medea, Algeria. Numerical analyses using Geo-Slope software are carried out under static, hydrological, and seismic conditions to quantify the improvement in stability and to assess the potential of this sustainable technic as an alternative to conventional reinforcement methods.

## Methodology

The methodology of this research is based on numerical approach aimed at evaluating the behavior of soil reinforced with tire chips under both static and seismic conditions. Laboratory tests (Atterberg limits, density, and Proctor compaction, direct shear) to determine specific geotechnical soil properties. These tests revealed the soil’s cohesion, internal friction angle, and optimum dry density. Tire chips with controlled dimensions were then incorporated into the soil at different percentages, mixed at the optimum moisture content, and compacted to achieve a dry density within ± 5% of the Proctor value. These parameters were implemented in a numerical model based on the Mohr–Coulomb failure criterion, considering three types of materials: marly clay, soft marl, and hard marl. Slope stability was evaluated using the safety factor method. This analysis covered both static and seismic situations. Regulations used include Eurocode 8 and the Algerian Seismic Code RPA 99 (2003).

## Presentation of study area

### Geographical location

**Fig. 1:**
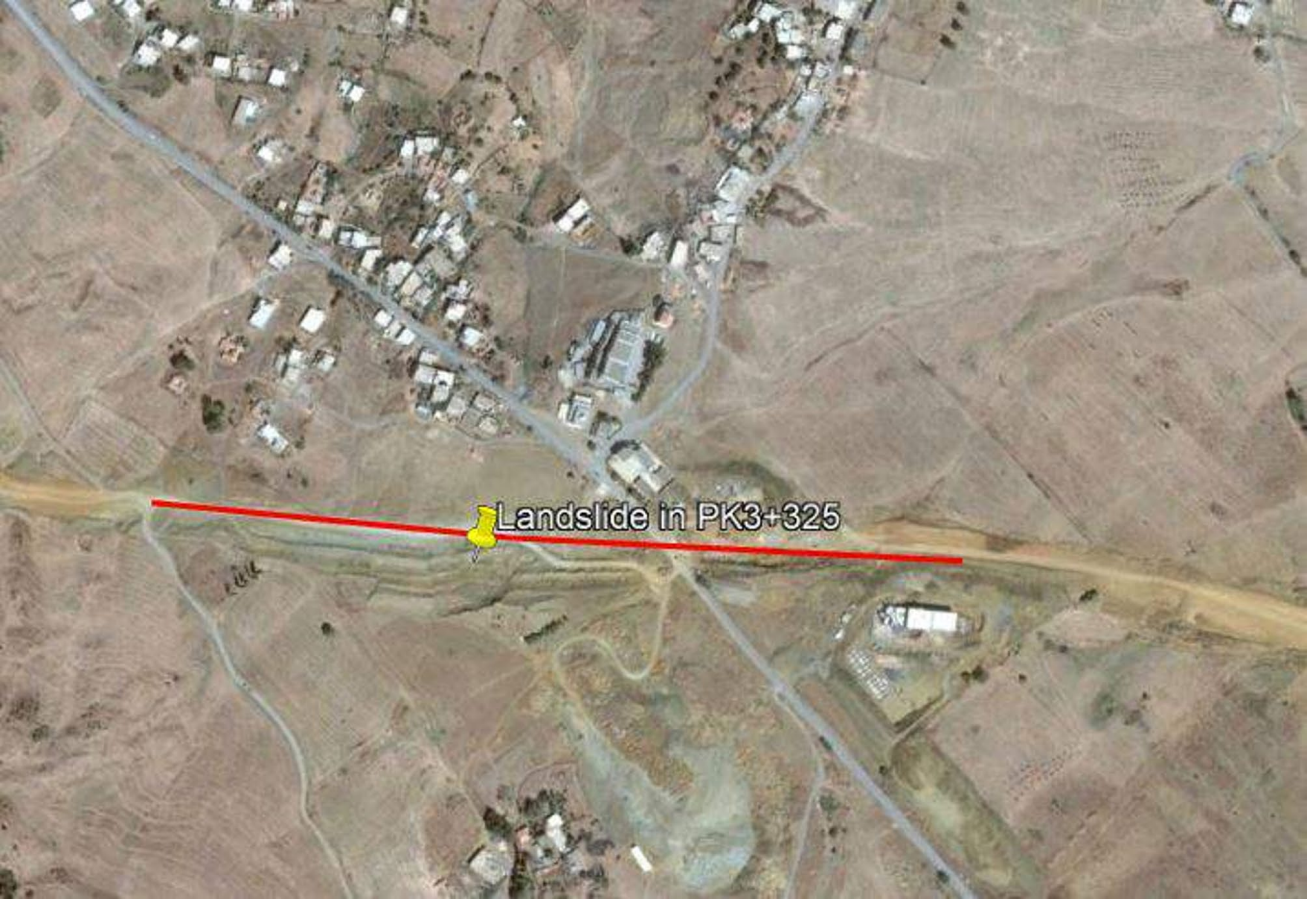
Satellite view of the road of landslide in Google Earth. (Version 7.3.6., https://www.google.com/earth/versions/).

The study area is part of the Draa Essamar commune, located 4 km west of the town of Medea, in a mountainous area at 806 m altitude. The landslide site is located on the upstream side of the roadway (embankment). From an environmental standpoint, the site is located in an area with no major population centers, surrounded by forested hills. Nevertheless, the road provides a link between the various villages. The general topography and alignment of the PK3 + 400 landslide are illustrated in Fig. 1, which shows the affected section along the roadway as captured from Google Earth.

### Geological and geotechnical setting

After consulting the BLIDA geological map at a scale of 1:200,000, it appears that the Medea region belongs to the Tellian Atlas mountain range and the northern end of the high plateaus. Cretaceous formations are generally found in the northern part and Cenozoic deposits in the southern part. The oldest rocks in the region are those of the Triassic period.

#### Lithostratigraphy (The Neogene)

Neogene deposits are represented by Miocene formations, with:

- Lower sandy, friable sandstones interspersed with pudding stones, mainly on the edges of the Medea basin. This layer, which varies greatly in thickness, reaches up to 80 to 100 m.In some places; these sandstones form the base of the Helvetian stage because they are closely related to the underlying clay layer. The layers of detritus represent fill deposits, and their rapid fluctuations can be monitored at narrowly spaced intervals.
- Marly clays are a distinctive feature of the Lower Helvetian landscape.
- Red gypsum clays, most often alluvial in appearance, occur in the Helvetian anticline.

#### Cretaceous formations

A thick layer comprising deposits from the Lower to Upper Cretaceous periods represents the Cretaceous formations.

Well-bedded bacillary limestones and marls are the representation of this mapped Lower Cretaceous formation ((C^5–4^)).

#### Tectonics overview

The western Medea region is characterized by folded and fractured Neogene structures, where anticline folds are particularly complicated. This tectonic framework allows the Cretaceous and Oligocene strata to outcrop and results in three synclinal zones, which present the complete Helvetian stage series with variable thicknesses. The region has also undergone Pliocene–Quaternary tectonics, which is still evolving today through recorded seismic activity. The Medea region falls under the Tellian domain and is distinguished by the Blida and Tablat anticlinoria, which correspond to autochthonous massifs bound by the Tellian nappes to the south. The recorded tectonic deformations, related to Alpine orogenesis, are subdivided into tangential deformations expressed by thrust sheet displacements, flexible deformations expressed by folding, and brittle deformations expressed by faulting in multiple directions. It can be concluded from this context that the study area is situated in a tectonically active and moderately unstable terrain. The stability of slopes and infrastructure in the study zone may be influenced by the persistence of seismic activity and structural complexity of the region.

### Landslide description

Ground movement upstream of the alignment (on the cut bank side) is caused by this landslide. The decompressed clays that make up the cut bank are affected by a toe slide.

The cross-section was cut back. The hard marl foundation provides a solid foundation for the roadway.

Water can infiltrate the embankment and roadbed because there are no concrete ditches or downspouts.

**Fig. 2:**
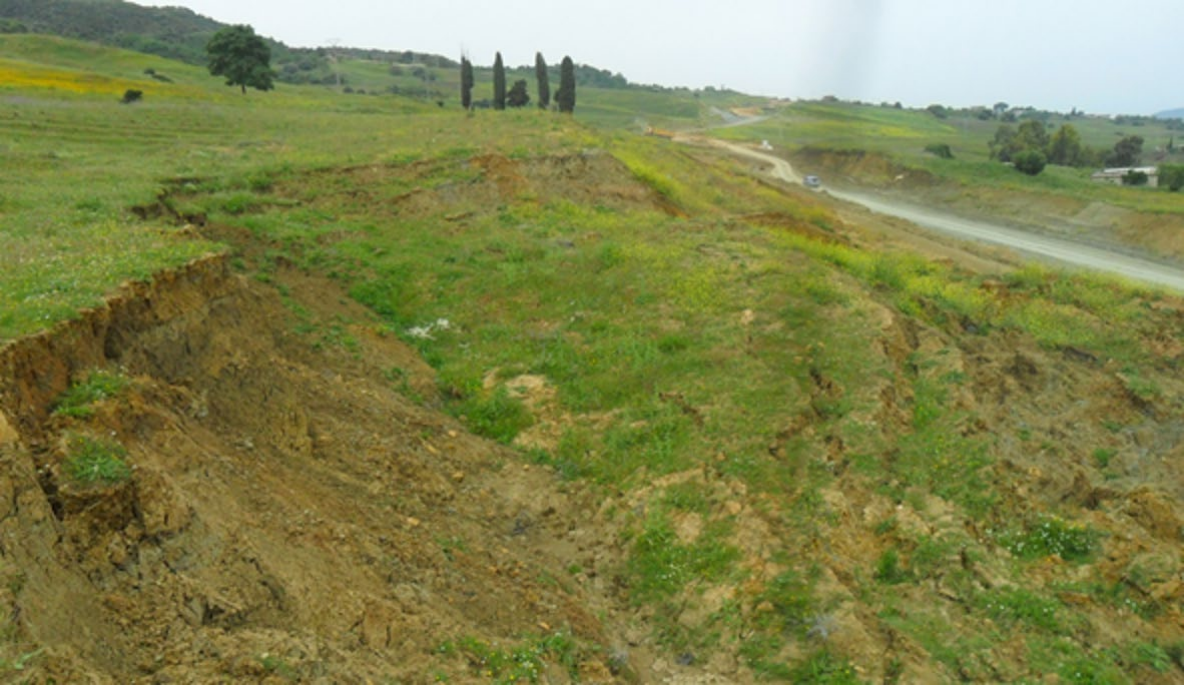
Decompressed state of the decompressed clays characterizing the embankment. (Field photograph taken by the authors, 2024).

The decompressed state of the marly clays forming the embankment is illustrated in Fig. 2, which highlights the visible cracking and sliding features observed on site.

#### Dimensions of the landslide

- The area affected by the landslide is 225 m long and up to 30 m high.
- The cracks are open in places, with scarping at the head of the cut slope.
- Open cracks at the heart of the cut slope, allowing water to infiltrate and thus developing interstitial pressures detrimental to overall stability.
- Embankment slope characterized by intense erosion
- Escarpment at the head of the cut slope.
- The decompressed state of the decompressed clays characterizes the cut slope.

#### Causes of slope failure

Geological, hydrological, mechanical, and anthropogenic factors combine to cause the landslide at PK3 + 400 in Medea. The slope has a geological structure that consists of pliable, decompressed clay layers over soft marl, which have low shear strength and are vulnerable to deformation during weather changes. The presence of multiple upstream water sources leads to elevated pore water pressures, which reduce effective stress and accelerate failure. Due to the absence of surface or subsurface drainage systems, the slope becomes more vulnerable to infiltration. The slope’s geometry is unfavorable due to its steep inclination and significant elevation divergence, which increases the driving forces relative to the resisting forces. The slope stability has been further reduced because of the removal of the downstream buttress, resulting in the elimination of passive resistance at the toe. The Medea region, which is classified as category IIa according to RPA 99, experiences additional dynamic loading that, when coupled with high rainfall and occasional snowmelt, can lead to failure. Due to these factors in synergy, the mechanical performance of the slope gradually deteriorates, making it vulnerable to both static and dynamic instability.

### Hydrogeological conditions

The hydrological regime of the study area plays a critical role in slope stability. The site is located in a mountainous region subject to relatively high annual rainfall, often exceeding 800 mm, with seasonal snow events that contribute to intermittent infiltration. The upper slope is fed by multiple natural springs, providing a continuous source of groundwater recharge. The clay matrix, now decompressed, creates pathways for infiltration. It shows low permeability initially but allows water saturation gradually through fissures and cracks visible in field examinations. Positive pore water pressures develop along potential slip surfaces. The Mohr–Coulomb failure criterion explains how this specific process weakens the shear strength of materials. The slope lacks specific drainage systems. This absence allows runoff and groundwater to saturate critical areas for prolonged periods. This condition, combined with the region’s seismic hazard, intensifies instability risks in saturated conditions. Given these factors, accurate modeling of slope behavior under both dry and saturated states is essential. The use of a fully saturated model in numerical simulations is justified for the rainy season scenario, though future studies should incorporate transient seepage analyses for improved accuracy.

### Seismic conditions

**Fig. 3:**
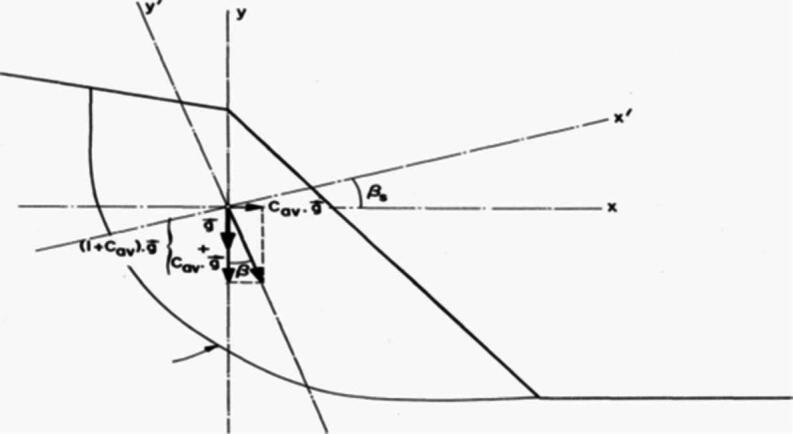
Simulation of an earthquake using the pseudo-static method.

The impact of an earthquake is handled using the “pseudo-static” method. Gravity changes with a horizontal acceleration coefficient (Cah) and a vertical acceleration coefficient (1 + Cav) in any direction. The operator provides these values (see Fig. 3).

According to the Algerian seismic code RPA 99 (Version 2003)^17,18^, the Medea region falls under category IIa, with a design ground acceleration of A = 0.15 g depending on the usage group. It should be noted that, in the absence of liquefiable soils, the stability verification can be carried out using an equivalent static analysis by applying the following seismic coefficient to all soil elements:

1 $${\text{Kh }} = \, 0.{\text{5 A }}\left( {\% {\text{ g}}} \right)$$

Coefficient (A) is the zone acceleration coefficient (table 4.1 of RPA 99) depending on the seismic zone and the importance group of the structure affected by the landslide. In our case, an acceleration of:

Kh = 0.5 (0.15) = 0.075.

Kv = 0.3, Kh = 0.022.

## Study of the Medea landslide using numerical modeling

The model will be modeled in 2D with a width of L = 142.37 m and a depth of H = 33,184 m.

**Fig. 4:**
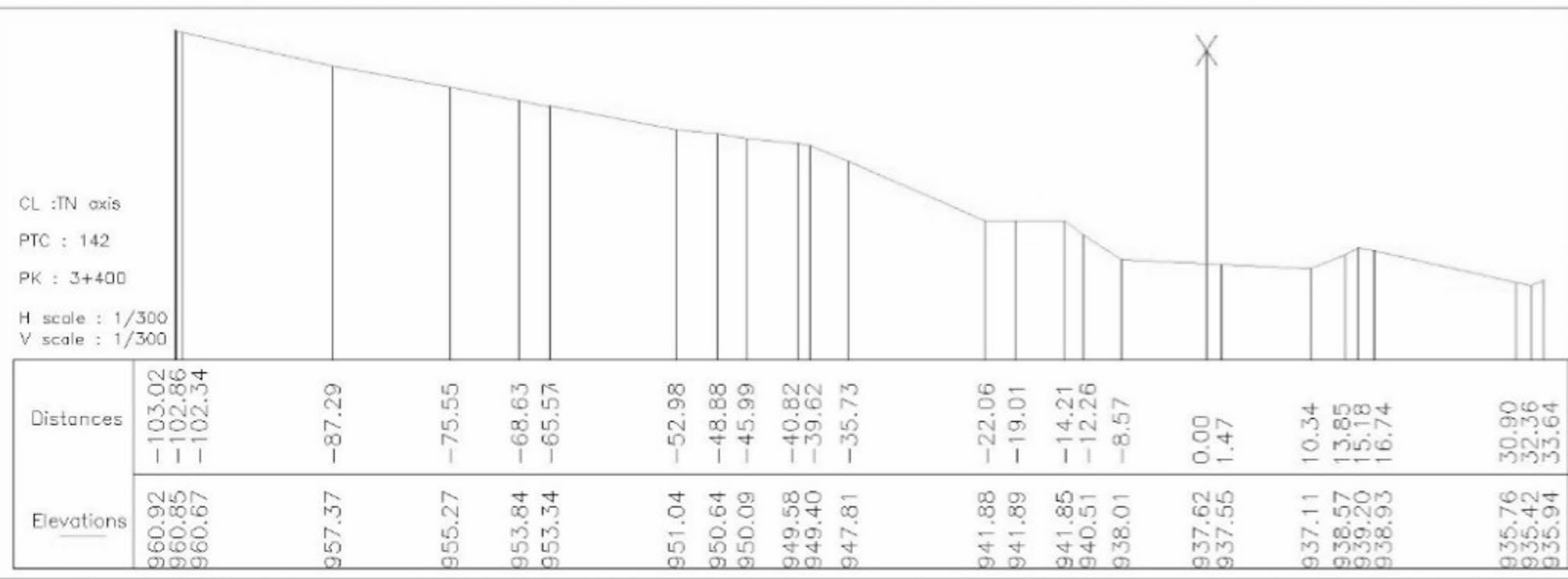
Ground profile adopted for modeling.

The geometry of the model is drawn on AutoCAD before being introduced into the Geo-slope (see Fig. 4).

### Geotechnical characterization of soils

The geotechnical parameters considered in the calculations are the results obtained from laboratory tests and crosschecks with dynamic petroleum tests, and they are summarized in Table 1.

**Table 1:** The geotechnical parameters of the ground.

Soil layer	γ (kN/m^3^)	Cohesion, c (kPa)	Friction angle, φ (°)	WL (%)	WP (%)	PI (%)
Marly clay	19	26	5.9	54	26	27
Soft marl	20	67	4,32	55	27	28
Hard marl (substratum)	20	67	10	69	31	36

### Slope stability analysis

#### Initial slope stability

The Factor of Safety (FoS) was calculated using the Simplified Bishop method under undrained (UU) conditions.

Table 2 presents the safety factors of the embankment at PK3 + 400 under three different loading conditions: dry (1.742), saturated (1.533), and seismic loading (1.296).

**Table 2:** Safety coefficients for the PK3 + 400 embankment under initial conditions.

Condition	FoS	Evaluation
Dry	1.742	Stable
Saturated	1.533	Marginally stable
Seismic load	1.296	Unstable

- Dry condition (FoS = 1.742)

**Fig. 5:**
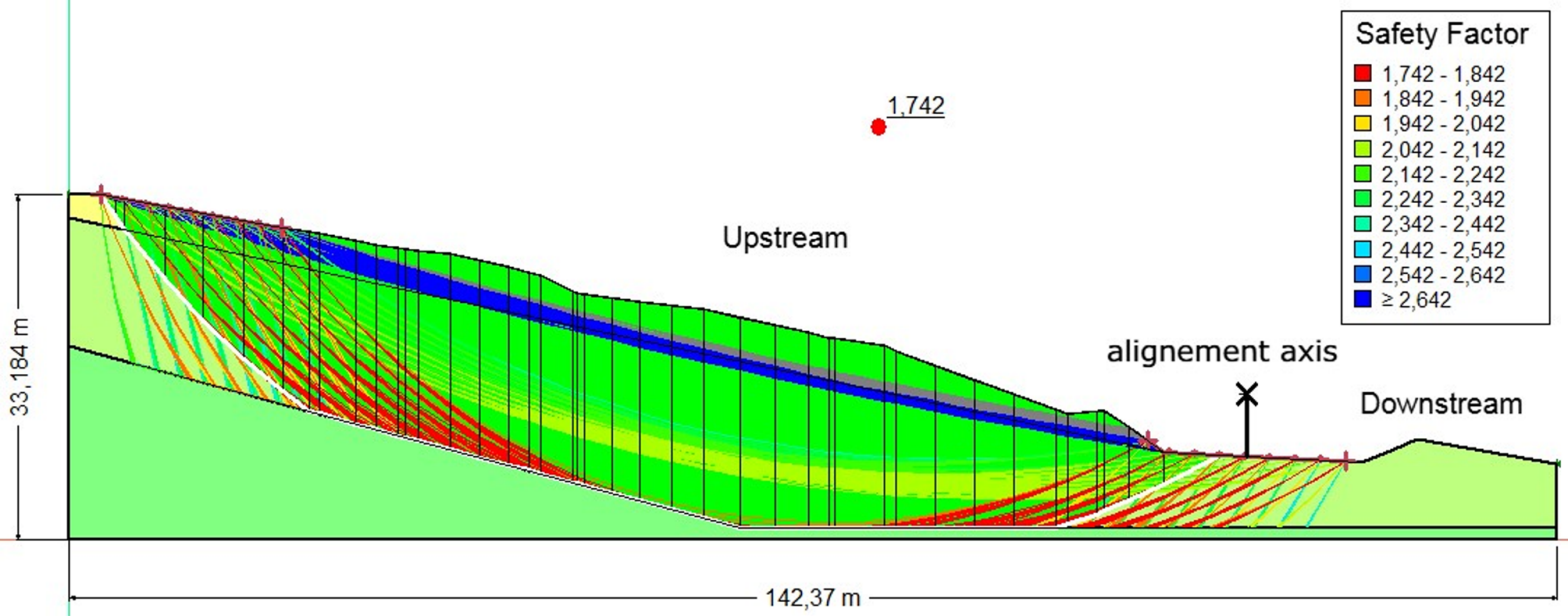
The safety coefficient obtained in the absence of a water table.

The slope is stable, with the FoS above the critical value of 1.5. In the absence of water, pore pressures remain negligible, and shear strength is fully mobilized through frictional and cohesive resistance (Fig. 5).

(b) Saturated condition (FoS = 1.533)

**Fig. 6:**
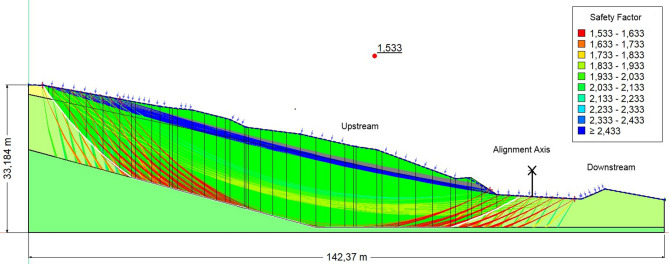
The safety coefficient obtained in the presence of water.

Water infiltration reduces stability by nearly 12% compared to the dry case. Increased pore pressures lower effective stress and shear strength, according to the Mohr–Coulomb criterion. The absence of drainage aggravates this effect, favoring progressive failure (Fig. 6).

(c) Seismic condition (FoS = 1.296)

**Fig. 7:**
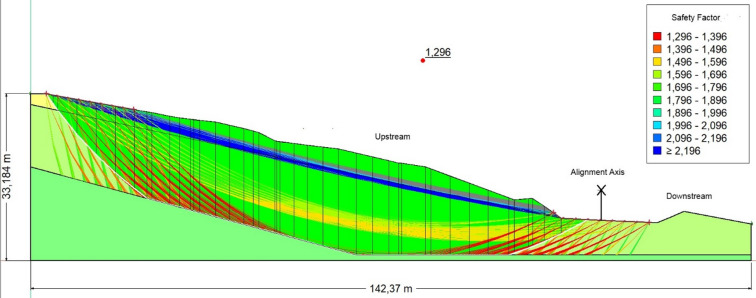
The safety coefficient obtained in the presence of seismic load and water.

Seismic loading reduces the FoS below 1.3, confirming clear instability. Although the slope is stable in dry and marginally stable in wet conditions, it becomes highly vulnerable to seismic action (Fig. 7).

### Influence of excavation

#### Stability analysis during earthworks (cut slope)

**Fig. 8:**
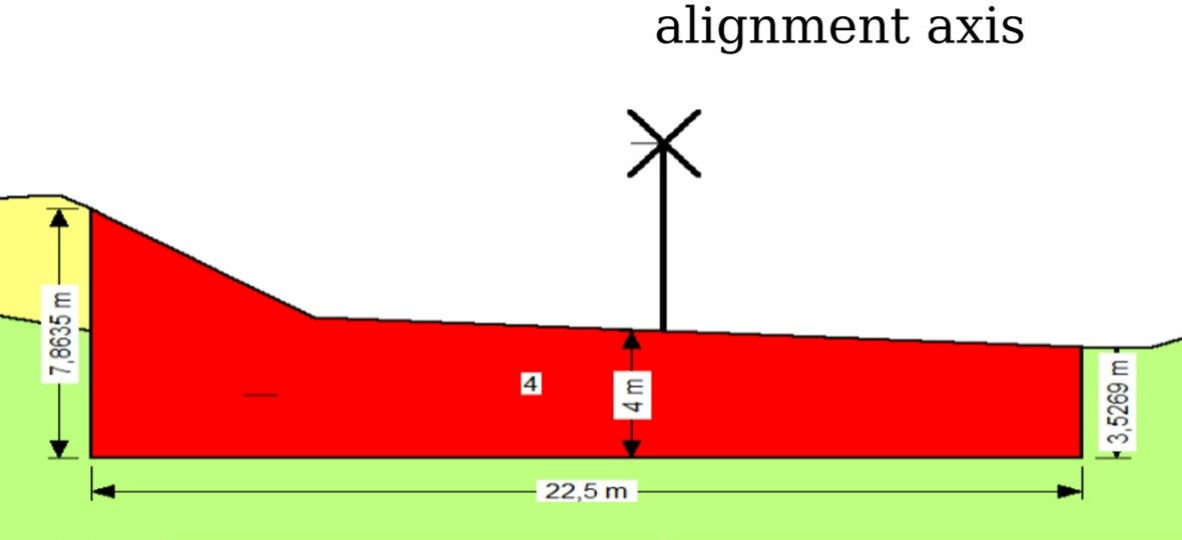
Dimensions of excavation (4 m).

Excavation depths of 1–4 m were modeled under seismic loading (Fig. 8).

#### Excavation stages with positive vertical seismic load

#### Interpretation

The FoS presented in Table 3 decreases progressively with depth, dropping by ~ 5.5% from 1 to 4 m. Vertical seismic acceleration amplifies shear stresses and reduces stability margins. Even shallow excavation (FoS = 1.115 at 1 m) is close to the critical seismic design range (1.1–1.2), highlighting the slope’s vulnerability. Water infiltration exacerbates structural instability, highlighting the essential need for reinforcement measures in seismic-prone areas such as Medea (classified as Zone IIa according to RPA 99 regulations).

**Table 3:** Evolution of the FoS with a positive vertical seismic load.

Excavation (m)	1	2	3	4
Safety coefficients (FOS)	1.115	1.094	1.074	1.054

#### Excavation stages with negative vertical seismic load

#### Interpretation

Under negative Kv, FoS presented in Table 4 decreases by ~ 5.3% between 1 and 4 m depth. Although values are slightly higher than in the positive Kv case, they remain well below the admissible limit (≥ 1.3–1.5). Negative vertical acceleration reduces the effect of gravity and partially mitigates destabilization, but instability persists at greater depths.

**Table 4:** Evolution of the FoS with a negative vertical seismic load.

Excavation (m)	1	2	3	4
Safety coefficients (FOS)	1.139	1.118	1.099	1.079

## Results and interpretation

The analyses reveal the following key points:

### Dry conditions

Without water infiltration, the slope remains stable above the required 1.5 threshold. This occurs because minimal pore water pressure enables the soil to utilize its complete shear strength from both cohesion and internal friction. The resisting forces clearly dominate over the driving forces, which explains why the slope is considered safe under dry conditions.

### Water saturation

When the soil becomes saturated, water infiltrates the pores, leading to an increase in pore water pressure. According to the Mohr–Coulomb failure criterion, it reduces the effective normal stress and therefore the available shear strength of the soil. Consequently, the FoS drops by about 12% compared to the dry case. Although still slightly above the critical threshold, this condition reflects marginal stability and highlights the destabilizing role of groundwater infiltration in slope failures.

### Seismic loading

Under seismic forces, additional dynamic stresses act on the slope mass. The pseudo-static approach accounts for horizontal and vertical seismic accelerations, which increase the driving forces along potential, slip surfaces. The safety factor falls below the critical limit of 1.3, indicating clear instability. This result demonstrates that the slope, already weakened by geological and hydrological conditions, becomes highly vulnerable to seismic excitation.

### Excavation effects

Excavation reduces lateral confinement and alters the stress distribution within the slope. As the excavation depth increases from 1 to 4 m, the FoS decreases progressively, reaching values close to 1.0, the threshold for imminent failure. The effect of vertical seismic loading (positive or negative Kv) further accelerates instability by modifying the balance between driving and resisting forces. It shows that deep excavation under seismic conditions substantially increases landslide risk, even when initial stability is acceptable.

### Critical interaction

The most dangerous scenario occurs when both water infiltration and seismic forces act simultaneously. Elevated pore water pressures reduce effective stresses, while seismic loading increases driving forces. This double effect causes the FoS to drop well below admissible values, confirming that the slope is unsafe without reinforcement. This combined scenario is typical of many real-world landslides, where rainfall and seismic activity often occur together, and producing catastrophic slope failures.

These results demonstrate that the PK3 + 400 slope is unsafe under saturated and seismic conditions, particularly during excavation. They also underline the essential role of drainage, hydrogeological control, and reinforcement strategies (e.g., tire–soil reinforcement) to ensure long-term stability.

## Slope reinforced using the tire-soil technic

The slope analysis revealed structural instability under saturated and seismic conditions, with FoS values consistently below 1.3. Without stabilization, excavation works present a significant risk of collapse. Tire-soil reinforcement was introduced as a potential solution, improving the soil’s cohesion and friction properties and thus raising the safety factor above admissible limits.

**Fig. 9:**
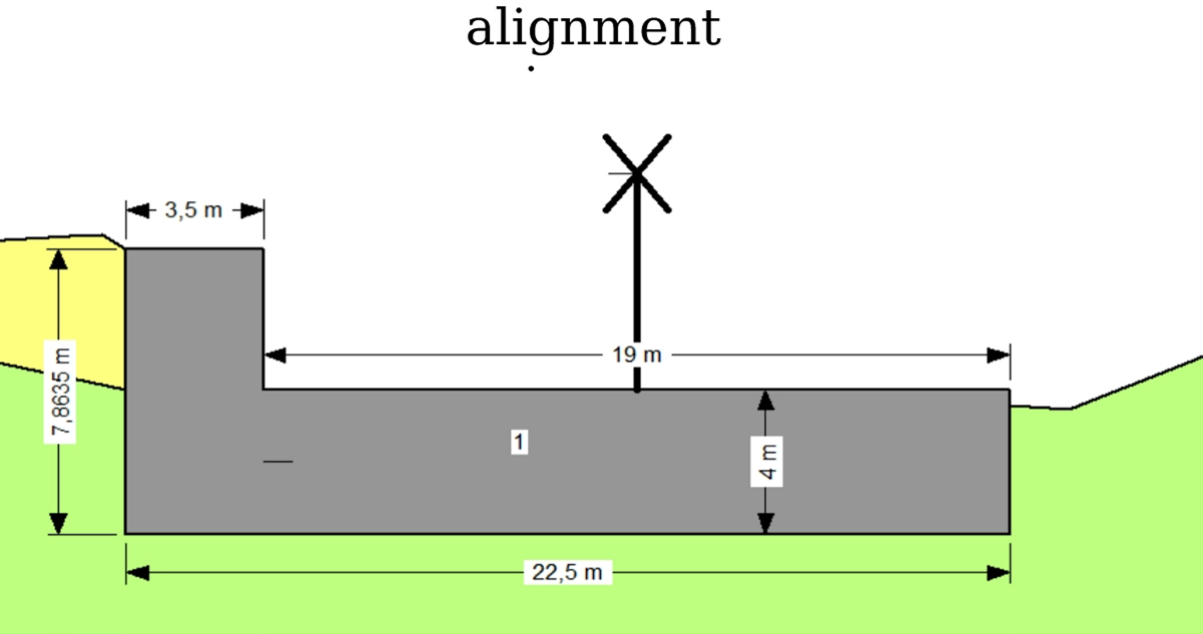
The dimensions of tire soil in 4 m depth.

The geometric configuration of the tire–soil reinforcement layers implemented at a 4 m depth is presented in Fig. 9.

### Material characteristics tire-soil

**Fig. 10:**
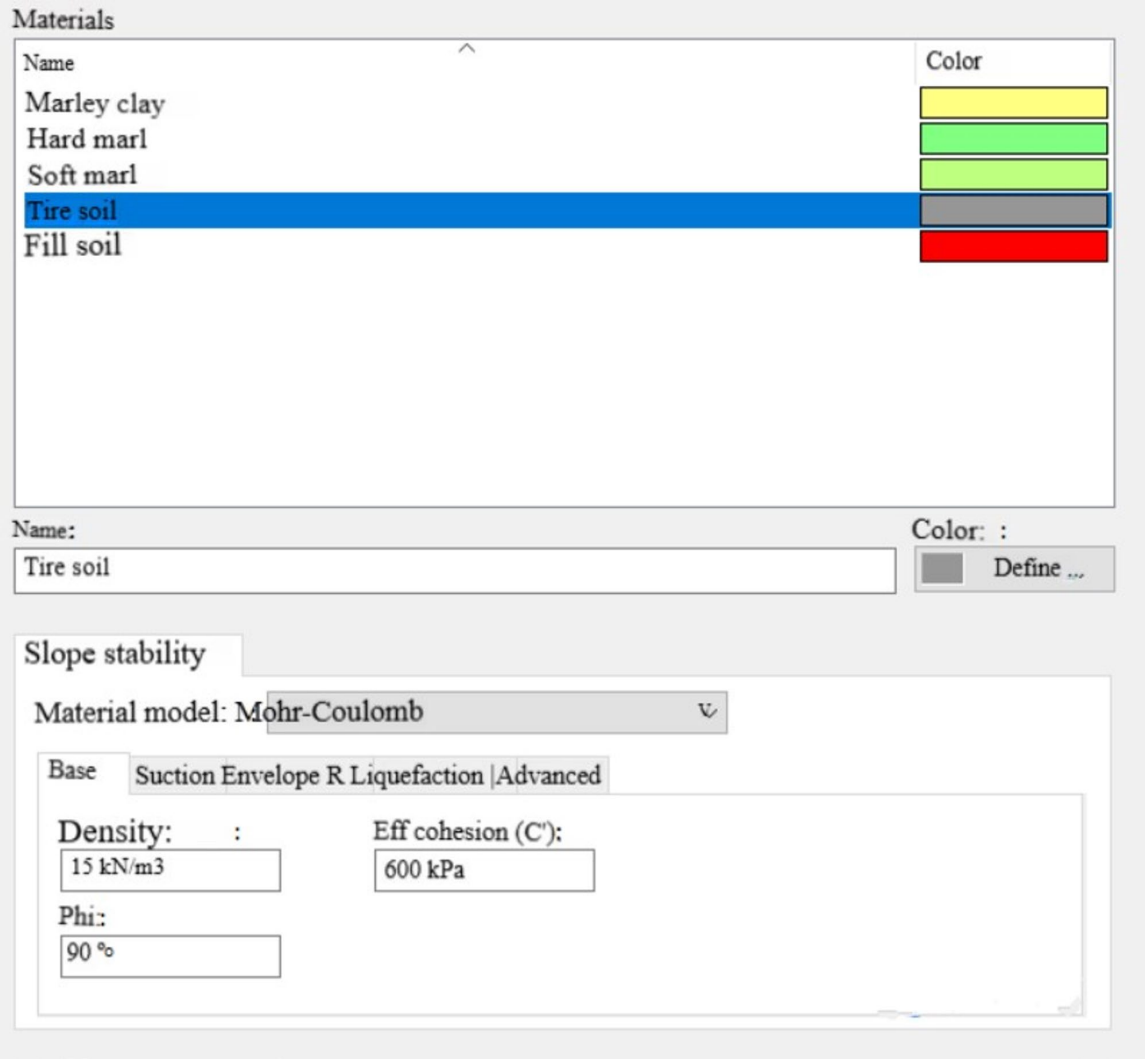
Geotechnical characteristics of tire-soil.

The mechanical parameters adopted for the tire–soil composite material were derived from previous studies by Nguyen Long (1985) and recent laboratory investigations on tire–soil mixtures. The specific geotechnical properties used in the numerical model are presented in Fig. 10, which summarizes the variation of shear strength and cohesion with tire content.

**Fig. 11:**
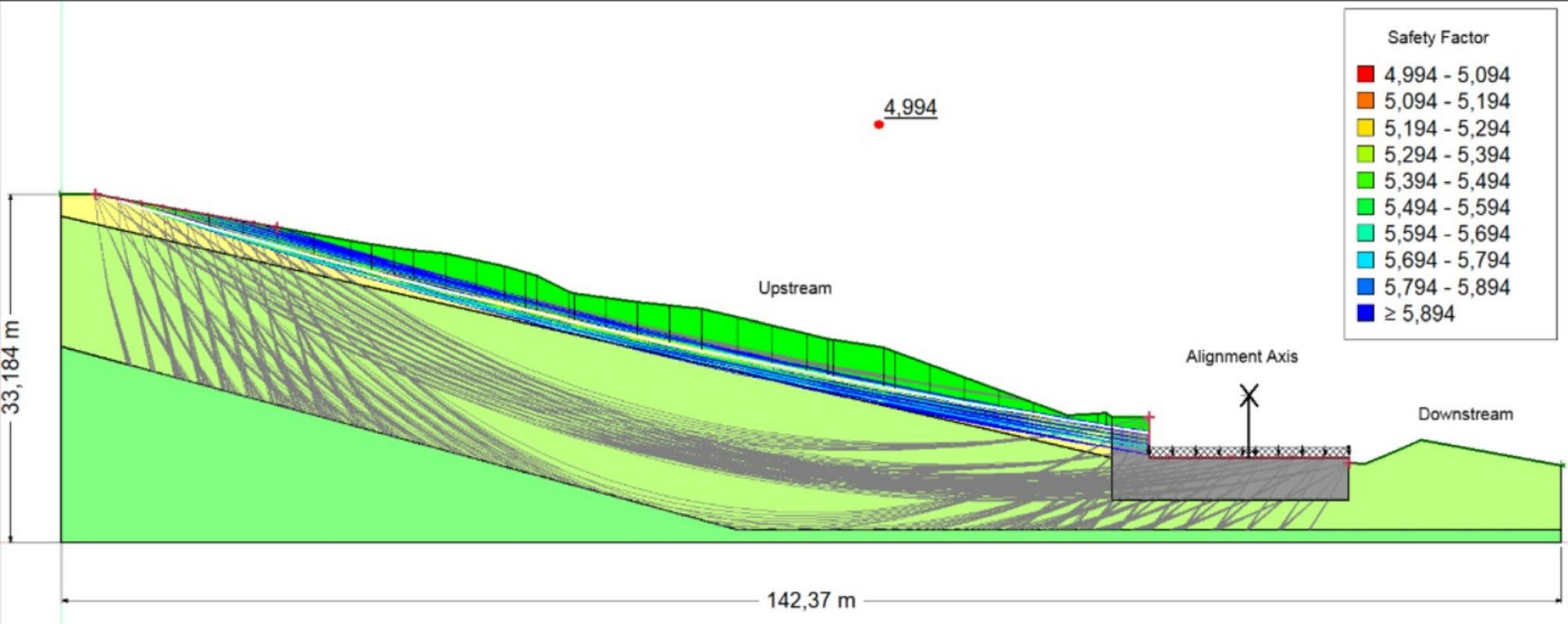
The safety coefficient obtained in the absence of a water table.

**Fig. 12:**
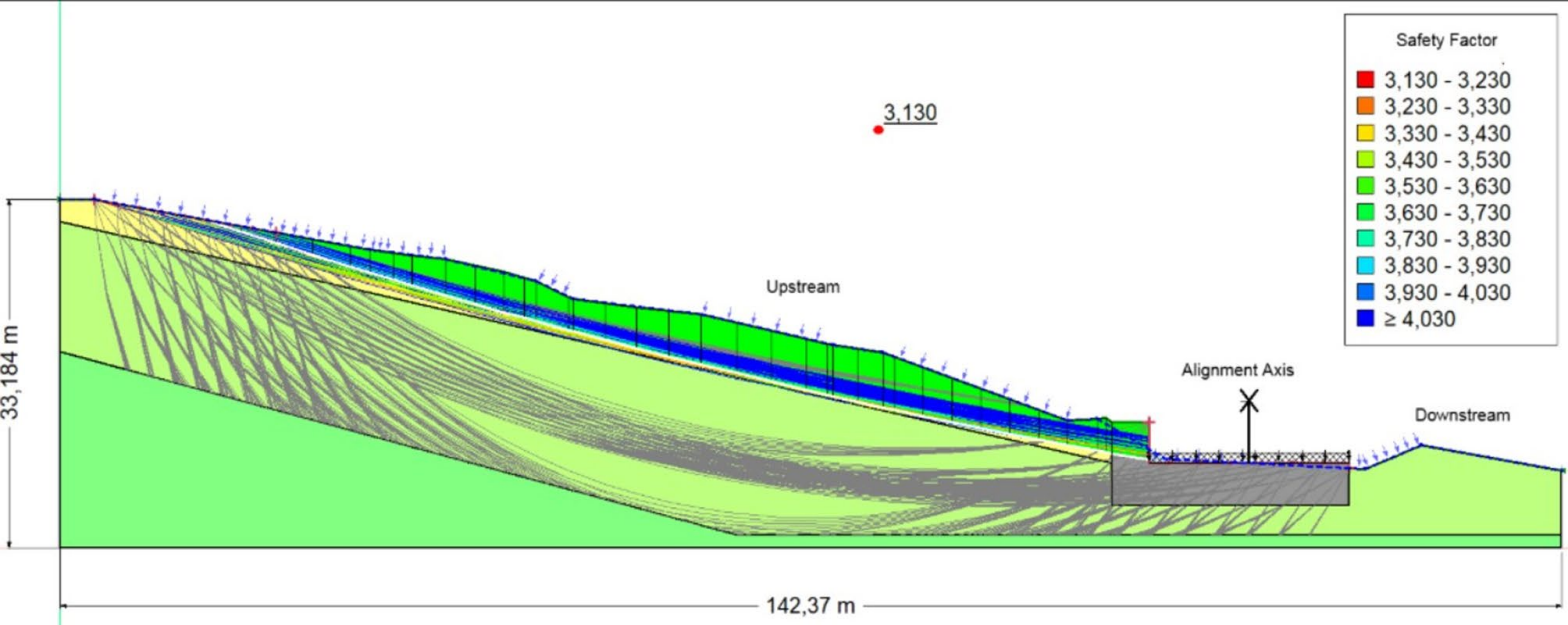
The safety coefficient obtained in the presence of a water table.

**Fig. 13:**
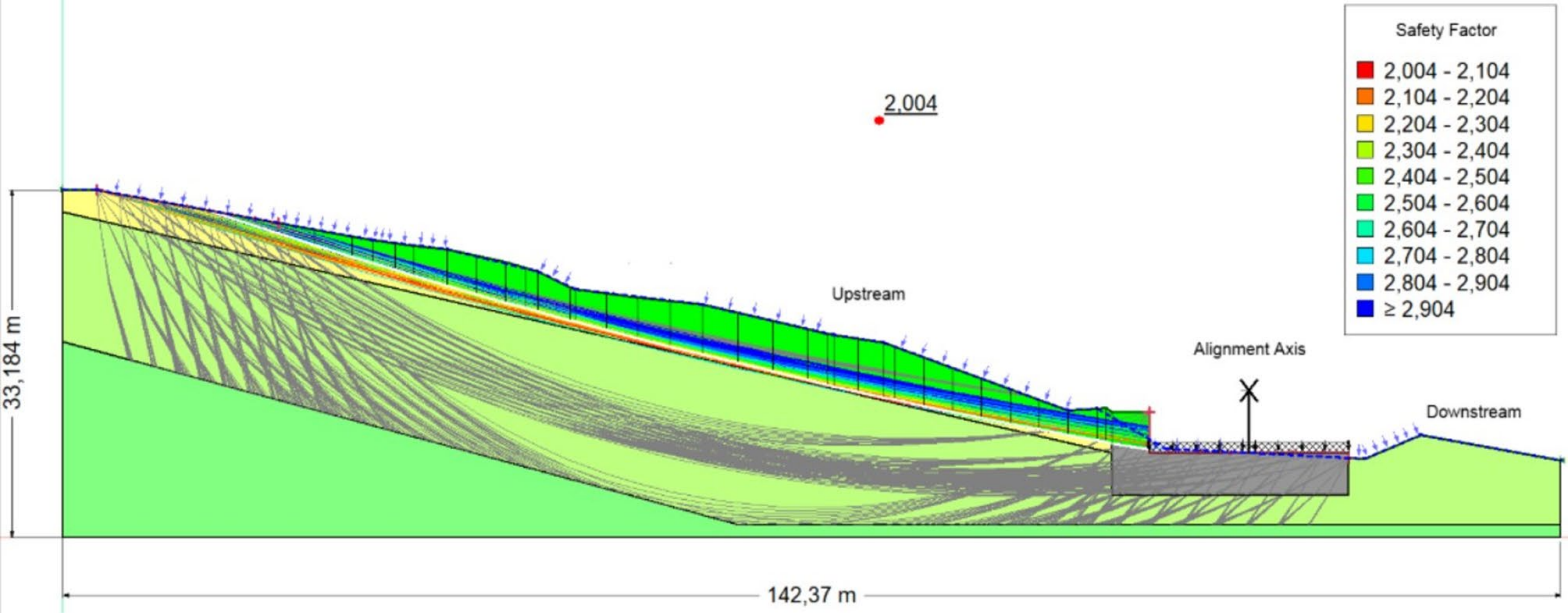
The safety coefficient obtained in the presence of seismic load and water.

The FoS is calculated for critical conditions after reinforcement installation:

Figures 11, 12, 13 Safety coefficients obtained with reinforcement in the scenarios: without water, with water, and with seismic load and water.

## Results and discussions

The Bishop Safety factor values for the four phases are given in the table below:

(The vertical seismic load is negative or positive).

Table 5 summarizes the Bishop Safety factor (Fs) values across four phases of excavation, considering both soil moisture conditions (dry vs. saturated) and the direction of vertical seismic loading (positive vs. negative). Across all phases, saturated conditions consistently reduce Fs by 15–30%, underlining the destabilizing effect of water infiltration.

**Table 5:** Bishop FoS values after reinforcement (four excavation phases, dry vs. saturated, positive vs. negative Kv).

Phase	Soil condition	Coeff, Kh	Coeff, kv	Fs
1	−	+	+	2.296
	−	+	−	2.354
	+	+	+	2.090
	+	+	−	2.138
2	−	+	+	2.296
	−	+	−	2.354
	+	+	+	2.090
	+	+	−	2.138
3	−	+	+	2.731
	−	+	−	2.791
	+	+	+	2.380
	+	+	−	2.433
4	−	+	+	3.089
	−	+	−	3.137
	+	+	+	2.004
	+	+	−	2.036

Key: Soil condition: (+) saturated, (−) dry.

(+) positive vertical seismic load, (−) negative vertical seismic load.

### Interpretation

- Phases 1–2: Stability remains modest, with Fs ranging from 2.090 (saturated, positive Kv) to 2.354 (dry, negative Kv). Groundwater infiltration significantly reduces stability, while positive vertical seismic loading amplifies driving forces.
- Phase 3: Noticeable improvement in stability, with Fs reaching 2.731–2.791 (dry) and 2.380–2.433 (saturated). Reinforcement counteracts destabilizing effects, although water continues to reduce Fs by ~ 0.35–0.40 compared to dry conditions.
- Phase 4: Maximum stability is achieved. In dry conditions, Fs exceeds 3.0, far above admissible thresholds. In saturated conditions, Fs remains above 2.0, acceptable but reflecting the need for drainage.

The tire-soil reinforcement ensures a safety coefficient exceeding 2.0, even under combined hydraulic and seismic conditions. Appropriate drainage infrastructure remains essential for preserving long-term stability.

### Stability analysis using different methods

The following table shows the values of the safety coefficients obtained in the last phase by the different methods available on the software.

#### Interpretation

The minimum factors of safety obtained from different analytical methods available in the Geo-Slope software are summarized in Table 6. All methods confirm slope stability with reinforcement (Fs > 1.5). The Janbu method yields the highest FoS (2.053), while Spencer gives the lowest (1.739). Differences are attributed to the assumptions of each method (moment vs. force equilibrium). Despite variations, all analyses validate the effectiveness of tire–soil reinforcement, especially at reinforcement depths of ~ 1 m. These findings emphasize how critical soil moisture levels and specific analytical methods are for proper slope stability assessments.

**Table 6:** Minimum FoS values obtained using different methods (final phase).

Methods	Valeur de Fs_min_
Bishop	2.004
Jumbo	2.053
Spencer	1.739
Morgenstern-price	1.915

### Comparison with literature

The findings of this study are consistent with previous research on tire–soil reinforcement.^14^ demonstrated that the incorporation of recycled tires improves shear strength and enhances slope stability, particularly in seismic regions. Similarly, Nguyen^16,17^ emphasized the dual benefits of this technique, combining mechanical reinforcement with improved drainage, which reduces pore pressures and ensures long-term stability. Belabdelouhab and Trouzine^15^ further confirmed its effectiveness in Algerian contexts, reporting both improved stability and significant environmental benefits through waste tire reuse. At position PK3 + 400, concrete data confirms our findings. The tire-soil reinforcement method increased safety coefficients from 1.296 to above 2.0 during earthquakes and wet conditions. This substantial enhancement ensures structural integrity even when subjected to exceptional stress factors. This confirms the technique’s dual role as both a reliable mechanical stabilization strategy and a sustainable geotechnical solution adapted to local soil and seismic characteristics.

## Conclusion

This study demonstrated that, while the PK3 + 400 slope in Medea, Algeria, is stable under dry conditions, it becomes unstable when saturated and under seismic loading. Applying the tire–soil reinforcement technique significantly improved stability, increasing shear strength and raising the safety factor above the critical threshold of 1.3, even when subjected to combined hydro-seismic stresses. The analysis confirmed the consistency of the results obtained using different stability methods (Bishop, Janbu, Spencer, and Morgenstern–Price), which highlights the reliability of this approach. Future research must examine soil parameter variations and test model assumptions. Beyond its engineering performance, tire–soil reinforcement offers notable environmental and economic benefits, such as recycling waste tires, reducing construction costs, and providing flexibility against dynamic loads. For lasting durability, prepare the site properly. Install reinforcement of adequate thickness (about 1 m). Ensure effective drainage. Conduct regular monitoring. These steps will guarantee successful implementation. Overall, tire–soil reinforcement has emerged as an innovative, sustainable, and efficient technique for slope stabilization, offering a viable alternative to traditional reinforcement methods.

## Data Availability

The datasets generated and analyzed during the current study are available from the corresponding author on reasonable request. All numerical modeling data and GEOSLOPE 2018 simulation files used in this work are archived and can be shared upon request for academic or research purposes. This declaration has been saved and submitted as part of our manuscript submission.

## Abbreviations

Fs: Factor of safety, representing the ratio between available resistance and the applied loading on the slope
UU: Undrained conditions, indicating saturated soil conditions where drainage is negligible during loading
Kh: Horizontal reinforcement coefficient applied to the soil using recycled tires
Kv: Vertical reinforcement coefficient applied to the soil using recycled tires
$${\varphi }_{i}$$: Friction angle
$$\gamma$$: Unit weight
C: Cohesion
Tire-soil reinforcement: A technique involving the integration of recycled tires into the soil to improve slope stability.
Bishop, Janbu, Spencer, and Morgenstern–Price: Analytical methods for slope stability analysis, each with specific assumptions regarding force and moment equilibrium

## References

[1] Ataei, M. & Bodaghabadi, S. Comprehensive analysis of slope stability and determination of stable slopes in the Chador–Malu iron ore mine using numerical and limit equilibrium methods. *J. China Univ. Min. Technol.* **18**, 488–493. 10.1016/S1006-1266(08)60281-3 (2008).

[2] Australian Geomechanics Society (AGS). Landslide risk management concepts and guidelines. *Austr. J. Emergency Manag.* **24**, 01 (2009).

[3] Chowdhury, R.N. & Flentje, P. Effective urban landslide hazard assessment. In *Proc. 8th Int. IAEG Congr.*, Vancouver, Canada, (1998).

[4] Chowdhury, R.N. & Flentje, P. Consideration of probability assessments relevant to hazard and risk for landslides. In *Proc. ICASP8 Conf.*, Sydney, Australia, (1999).

[5] Chowdhury, R. N. & Flentje, P. Role of slope reliability analysis in landslide risk management. *Bull. Eng. Geol. Env.* **62**, 41–46. 10.1007/s10064-002-0166-1 (2003).

[6] Janbu, N. Slope stability computations In Embankment-dam Engineering. In *Textbook* (eds Hirschfeld, R. C. & Poulos, S. J.) 1973 (Wiley, NY, 1973).

[7] *Int. J. R. Mech. Mining Sci. Geomech. Abstracts* **12**, 67, 10.1016/0148-9062%2875%2990139-4 (1975).

[8] Morgenstern, N. R. & Price, V. E. The analysis of stability of general slip surface. *Geotechnique* **15**, 79–93. 10.1680/geot.1965.15.1.79 (1965).

[9] Sarma, S. K. A note on the stability of slopes. *Geotechnique* **37**, 107–111. 10.1680/geot.1987.37.1.107 (1987).

[10] Helexa, M. et al. Comparison of radial ply and cross ply tire in terms of achieved rolling resistance and soil compaction in a soil test channel. *MDPI.* **15**(8), 1397. 10.3390/f15081397 (1937).

[11] Zeng, H., Zhao, C., Chen, S. & Zang, M. Numerical simulations of tire-soil interactions: A comprehensive review. *Arch. Comput. Methods Eng.* **30**, 4801–4829. 10.1007/s11831-023-09961-6 (2023).

[12] Thorpe, D. F. et al. Impacts of bulk density and water content on the tire–soil contact zone of agricultural vehicles. *Soil Tillage Res.* **245**, 105–672. 10.4025/actasciagron.v46i1.67906 (2024).

[13] Golanbari, B. & Aref, M. An analytical model for stress estimation at the soil–tire interface using the dynamic contact length. *J. Terramech.* **111**, 45–57. 10.1016/j.jterra.2023.08.006 (2023).

[14] Tekeste, M. Z., Way, T. R., Birkenholz, W. & Brodbeck, S. Effect of increased deflection tire technology on soil compaction. *Trans. ASABE* **66**, 75–84. 10.13031/ja.14794 (2023).

[15] Long, N.T. *Tires: Research, Development and Outlook*. PhD Thesis, INSA Lyon (1993).

[16] Belabdelouhab, F. & Trouzine, H., Tire–Soil in Algeria: Research, Construction Projects, and Environmental Protection. *Proceedings of the IVth International Congress on Renewable Energy and the Environment*, Tunisia (2009).

[17] Long, N.T. *Tire-soil*. Laboratory report, Department of Urban Planning, Housing, and Transportation, (1985).

[18] CGS (2003). Algerian Seismic Design Code, RPA 99 (Version 2003). Algiers, Algeria: National Applied Research Center in Earthquake Engineering (CGS), Ministry of Housing and Urban Planning.

[19] Kumar, S., Choudhary, S. S. & Burman, A. The effect of slope height and angle on the safety factor and modes of failure of 3D slopes analysis using limit equilibrium method. *Beni Suef Univ. J. Basic Appl. Sci.* **12**, 84. 10.1186/s43088-023-00423-3 (2023).

[20] Int. Conf. on Creative and Innovative Solutions in Civil Engineering (CISCE-2023). Jaipur, India, 11–12 August 2023. *IOP Conf. Ser. Earth Environ. Sci.* **1326**, 012117 , 10.1088/1755-1315/1326/1/012117 (2023).

